# Supplementary material for: Chemical Composition, Antioxidant, and Antimicrobial Activity of Dracocephalum moldavica L. Essential Oil and Hydrolate
Source: Plants (Basel). 2022 Mar 31;11(7):941. doi: 10.3390/plants11070941 (PMC9002726; doi:10.3390/plants11070941)
Supplement: Supplementary file 1 [file plants-11-00941-s001.zip › Table S1.pdf]

Supplementary Table S1. The "goodness of fit" kinetics models

Control		Quality parameters									
		χ^2	RMSE	MBE	MPE	r^2	Skew	Kurt	Mean	StDev	Var
S. aureus		0.01	0.07	0.00	0.71	0.99	-0.40	2.54	0.00	0.07	0.01
L. monocytogenes		0.01	0.09	0.00	1.17	0.99	0.03	-0.85	0.00	0.09	0.01
E. coli		0.01	0.09	0.00	0.86	0.99	-0.42	0.22	0.00	0.10	0.01
S. Typhimurium		0.01	0.08	0.00	1.06	0.99	-0.39	-1.17	0.00	0.09	0.01
**DMEO**	**Concentation**	χ^2	**RMSE**	**MBE**	**MPE**	r^2	**Skew**	**Kurt**	**Mean**	**StDev**	**Var**
S. aureus	MIC	0.14	0.28	-0.02	2.93	0.99	-0.20	3.14	-0.02	0.30	0.09
	2×MIC	0.00	0.00	0.00	0.00	1.00	-3.00	9.00	0.00	0.00	0.00
	4×MIC	0.00	0.00	0.00	0.00	1.00	-3.00	9.00	0.00	0.00	0.00
L. monocytogenes	MIC	0.21	0.34	-0.06	6.69	0.97	-0.45	-1.57	-0.06	0.35	0.13
	2×MIC	0.18	0.31	-0.06	7.52	0.98	1.19	2.17	-0.06	0.33	0.11
	4×MIC	0.00	0.00	0.00	0.00	1.00	-3.00	9.00	0.00	0.00	0.00
E. coli	MIC	0.14	0.28	0.03	14.54	0.98	-1.34	1.39	0.03	0.30	0.09
	2×MIC	0.24	0.36	-0.05	12.24	0.97	0.18	-0.80	-0.05	0.38	0.15
	4×MIC	0.46	0.50	-0.09	7.04	0.93	1.29	2.37	-0.09	0.52	0.28
S. Typhimurium	MIC	0.04	0.15	-0.03	2.23	1.00	-0.11	0.15	-0.03	0.15	0.02
	2×MIC	0.14	0.28	-0.02	2.93	0.99	-0.20	3.14	-0.02	0.30	0.09
	4×MIC	0.09	0.22	-0.04	5.25	0.98	1.69	4.47	-0.04	0.23	0.05
**DMH**	**Concentation**	χ^2	**RMSE**	**MBE**	**MPE**	r^2	**Skew**	**Kurt**	**Mean**	**StDev**	**Var**
S. aureus	MIC	0.05	0.17	-0.01	7.75	0.99	-0.61	0.91	-0.01	0.18	0.03
	2×MIC	0.17	0.31	-0.01	12.47	0.98	-0.30	-1.61	-0.01	0.32	0.10
	4×MIC	0.14	0.28	-0.05	9.10	0.98	0.67	1.32	-0.05	0.29	0.08
L. monocytogenes	MIC	0.01	0.07	0.00	8.67	1.00	-0.45	-0.37	0.00	0.07	0.01
	2×MIC	0.10	0.24	0.05	18.29	0.99	-0.70	0.22	0.05	0.24	0.06
	4×MIC	0.05	0.17	-0.03	4.39	0.99	0.17	0.01	-0.03	0.18	0.03
E. coli	MIC	0.26	0.38	-0.06	24.70	0.95	0.27	-1.12	-0.06	0.40	0.16
	2×MIC	0.24	0.37	-0.06	27.80	0.96	0.54	-0.89	-0.06	0.38	0.15
	4×MIC	0.20	0.33	-0.05	31.00	0.96	0.69	-1.04	-0.05	0.35	0.12

* χ^2 – reduced chi-square; RMSE – root mean square error; MBE – mean bias error; r^2 – coefficient of determination; Skew – skewness; Kurt – kurtosis; Mean – mean of the residuals; StDev – standard deviation of the residuals; Var – variance of the residuals
