# Supplementary figures and images for: Chemical Composition, Antioxidant, and Antimicrobial Activity of Dracocephalum moldavica L. Essential Oil and Hydrolate
Source: Plants (Basel). 2022 Mar 31;11(7):941. doi: 10.3390/plants11070941 (PMC9002726; doi:10.3390/plants11070941)

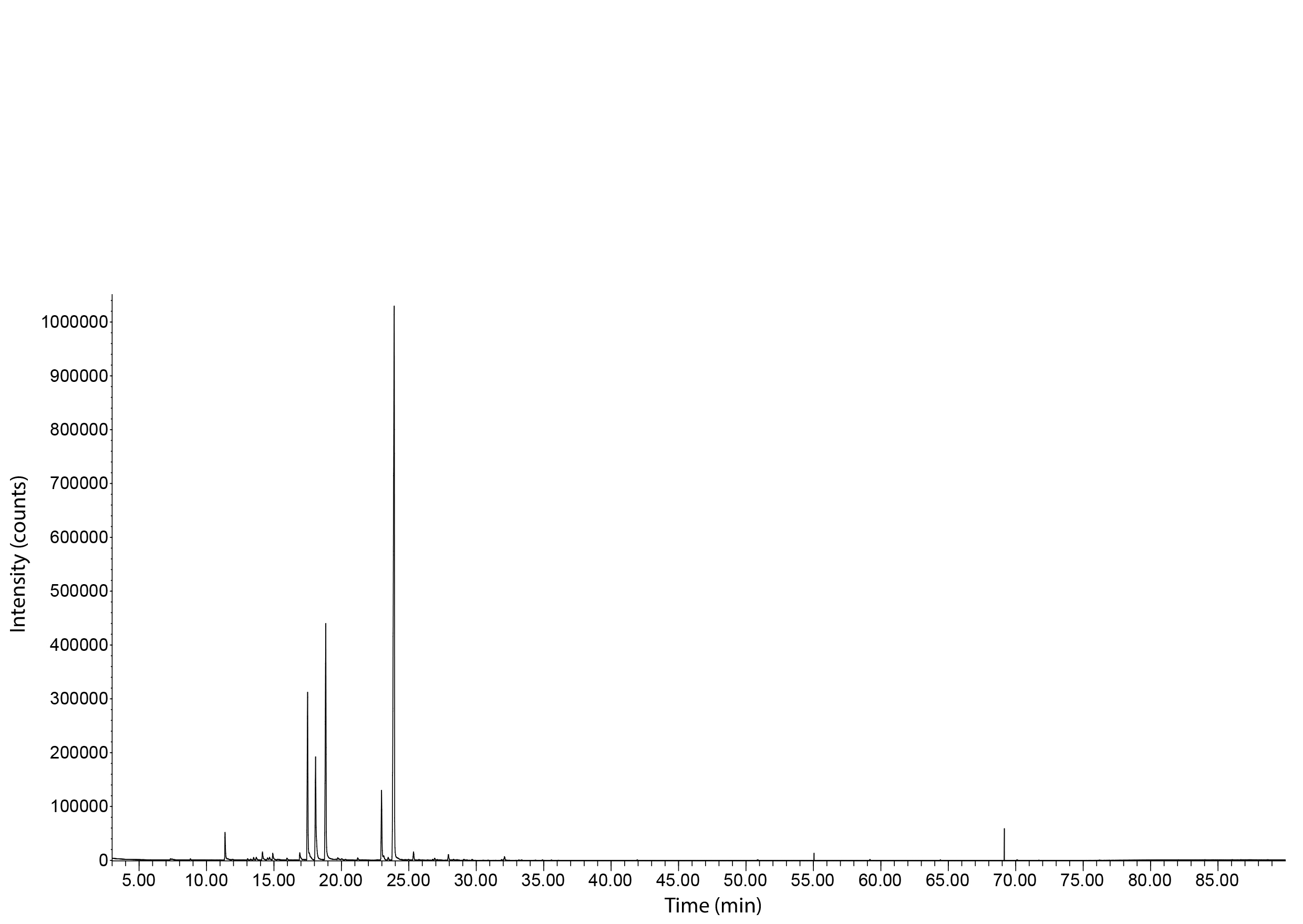

Supplement: Supplementary file 1 [file plants-11-00941-s001.zip › Figure S1.jpg]

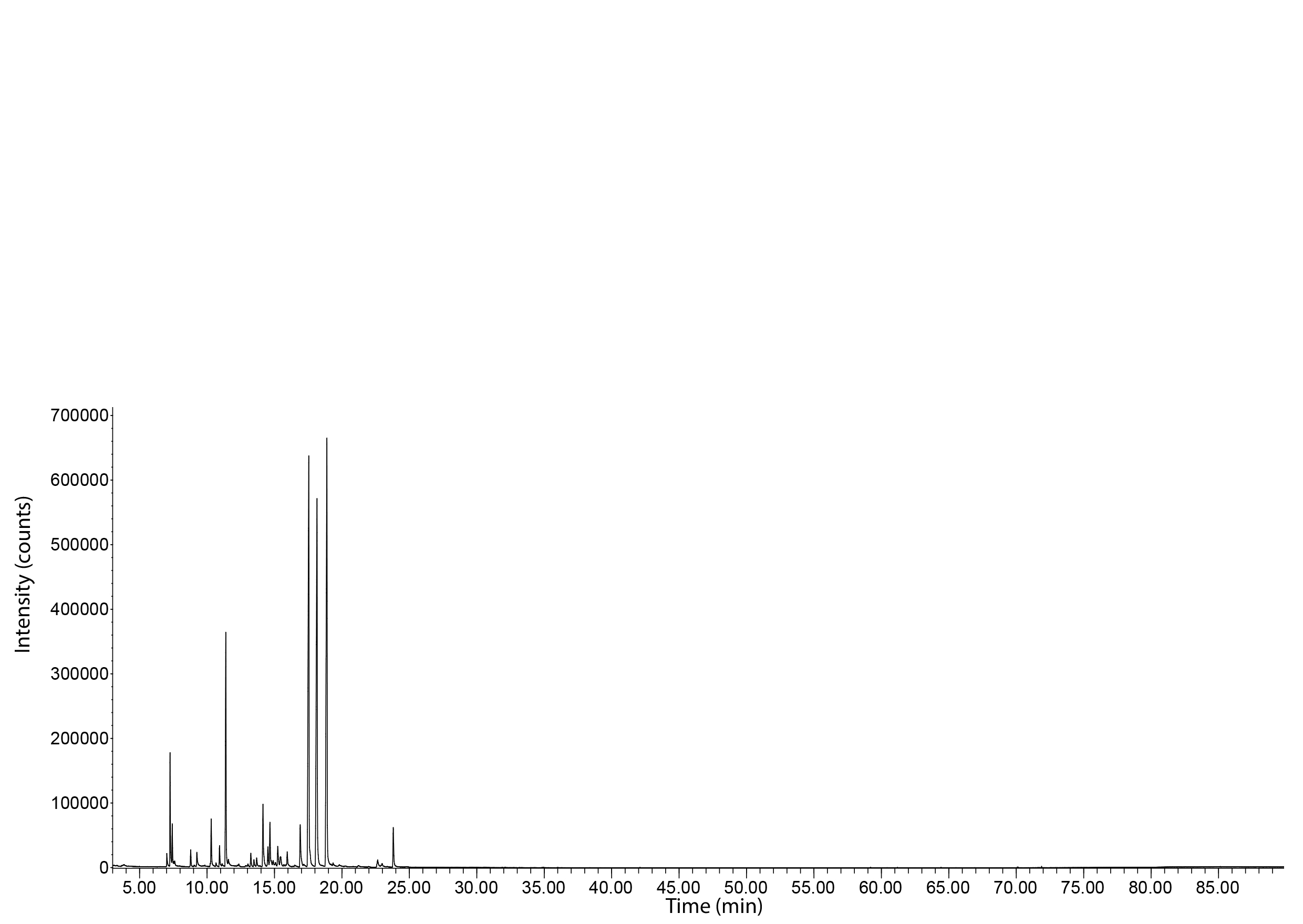

Supplement: Supplementary file 1 [file plants-11-00941-s001.zip › Figure S2.jpg]
